# Notch signals modulate *lgl* mediated tumorigenesis by the activation of JNK signaling

**DOI:** 10.1186/s13104-018-3350-5

**Published:** 2018-04-16

**Authors:** Maimuna Sali Paul, Ankita Singh, Debdeep Dutta, Mousumi Mutsuddi, Ashim Mukherjee

**Affiliations:** Department of Molecular and Human Genetics, Banaras Hindu University, Varanasi, 221 005 India

**Keywords:** Notch, *lgl*, *Drosophila*, Tumor overgrowth, JNK signaling, Cell death

## Abstract

**Objectives:**

Oncogenic potential of Notch signaling and its cooperation with other factors to affect proliferation are widely established. Notch exhibits a cooperative effect with loss of a cell polarity gene, *scribble* to induce neoplastic overgrowth. Oncogenic Ras also show cooperative effect with loss of cell polarity genes such as *scribble* (*scrib*), *lethal giant larvae* (*lgl*) and *discs large* to induce neoplastic overgrowth and invasion. Our study aims at assessing the cooperation of activated Notch with loss of function of *lgl* in tumor overgrowth, and the mode of JNK signaling activation in this context.

**Results:**

In the present study, we use *Drosophila* as an in vivo model to show the synergy between activated Notch (*N*^*act*^) and loss of function of *lgl* (*lgl*-*IR*) in tumor progression. Coexpression of *N*^*act*^ and *lgl*-*IR* results in massive tumor overgrowth and displays hallmarks of cancer, such as MMP1 upregulation and loss of epithelial integrity. We further show activation of JNK signaling and upregulation of its receptor, Grindelwald in *N*^*act*^*/lgl*-*IR* tumor. In contrast to previously described *Notch*^*act*^*/scrib*^−*/*−^ tumor, our experiments in *N*^*act*^*/lgl*-*IR* tumor showed the presence of dying cells along with tumorous overgrowth.

**Electronic supplementary material:**

The online version of this article (10.1186/s13104-018-3350-5) contains supplementary material, which is available to authorized users.

## Introduction

In the past decade, a keen interest has been shown to explore the oncogenic cooperation with loss of cell polarity in tumor progression and malignancy. Studies in *Drosophila* have revealed that the oncogenic form of Ras cooperates with loss of tumor suppressors, namely *scrib*, *lgl* and *dlg* to cause tumor cell invasion [1, 2]. The oncogenic form of Notch has also shown to cooperate with *scrib*^−*/*−^ to induce neoplastic overgrowth [2]. The loss-of-function mutation of Scribble complex genes (*scrib*, *lgl* and *dlg*) results in disruption of epithelial integrity followed by neoplastic tissue hyperproliferation [3–5]. However, the tumor formation caused by loss of *scrib, lgl* and *dlg* has been found to be restricted by the compensatory JNK mediated apoptosis [2, 6–8]. Among the Scrib complex genes, *lgl* was the first neoplastic tumor suppressor gene described in *Drosophila* [9]. The phenotypes of *lgl* mutant tissues show close similarity with that of the human epithelial cancers [10–12]. Although it has been shown that Notch cooperates with *scrib*^−*/*−^ to induce neoplastic growth, it is still unknown whether Notch works in the same way with loss-of-function of *lgl* also. Recently, Lgl has been shown to regulate Notch signaling via endocytosis [13]. However, it gives no substantial evidence on coupling of *lgl*-Notch effect on tumorigenesis. In the present study, we checked the effect of a tumor suppressor gene mutation, *lgl*, in activated Notch background, and found that *lgl* downregulation synergizes with activated Notch to induce overgrowth and migratory behavior. Here, we show that *N*^*act*^*/lgl*-*IR* tissues display the hallmarks of tumor overgrowth. Moreover, our study revealed that the effect of *N*^*act*^*/lgl*-*IR* tumor is mediated by the activation of JNK signaling through the upregulation of its receptor, Grindelwald.

## Main text

### Methods

Detailed description of methods used in this study is provided in Additional file 1.

### Results

#### Oncogenic Notch synergizes with RNAi mediated downregulation of *lgl* to promote tissue overgrowth

**Fig. 1:**
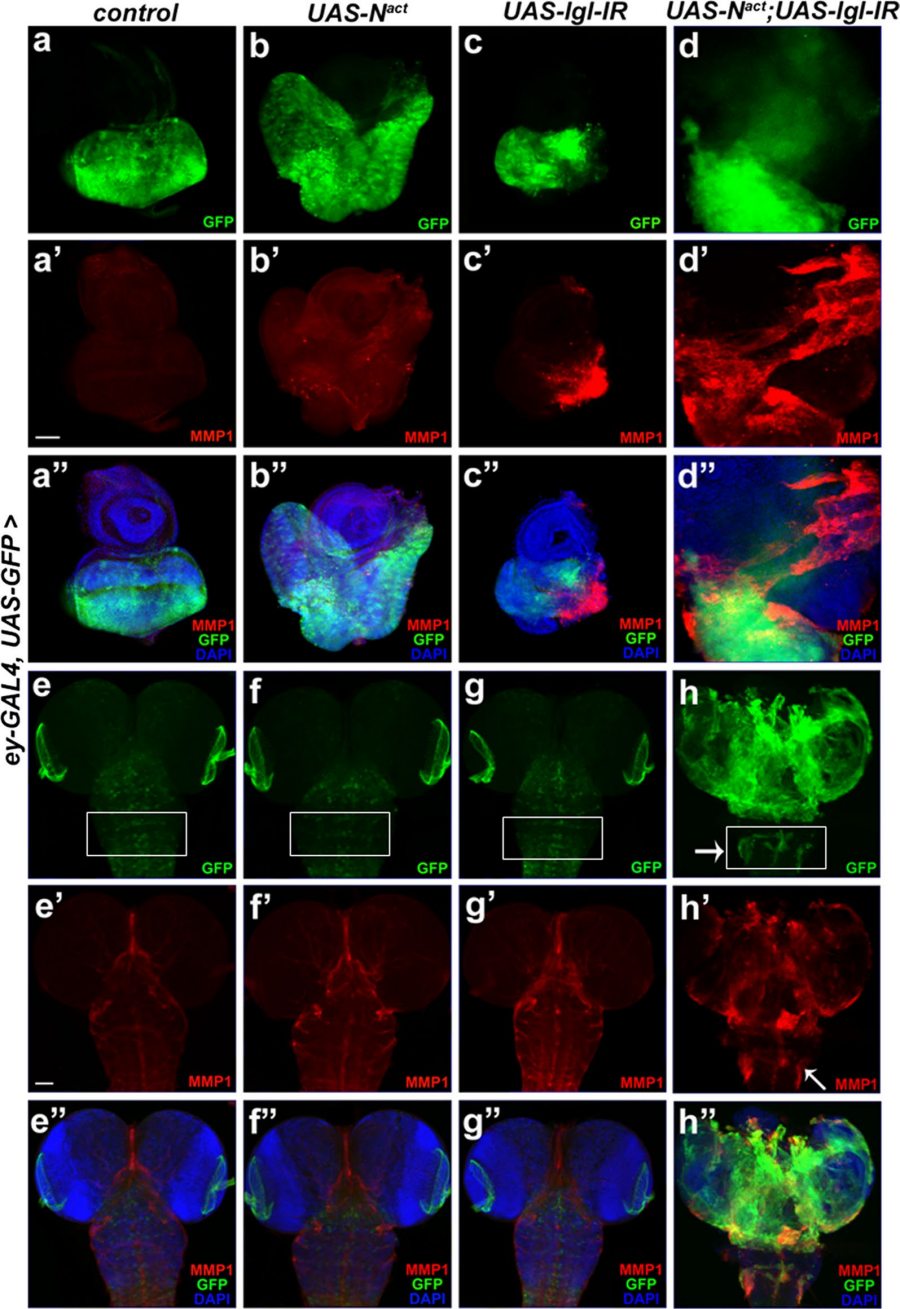
Oncogenic Notch synergizes with *lgl*-*IR* to induce tissue overgrowth. Fluorescent micrographs of eye imaginal discs and larval brains are shown. *ey*-*GAL4/*+ (control) eye imaginal disc shows expression of GFP (**a**) and MMP1 (**a**′). *ey*-*GAL4* driven *UAS*-*N*^*act*^ results in enlarged disc size (**b**) and shows slight enhancement in MMP1 expression (**b**′). *UAS*-*lgl*-*IR* eye disc driven by *ey*-*GAL4* (**c**) induces MMP1 expression (**c**′). *UAS*-*N*^*act*^ coexpressed with *UAS*-*lgl*-*IR* results in massively overgrown eye discs (**d**) and significant enhancement in MMP1 expression throughout the tissue (**d**′) compared to that of only *lgl*-*IR* (**c**′) or only *Notch*^*act*^ (**b**′) overexpressed eye discs. Images **a**″–**d**″ are merges of those in **a**–**a**′, **b**–**b**′, **c**–**c**′ and **d**–**d**′ with DAPI, respectively. Expression of GFP and MMP1 in brains of *ey*-*GAL4* driven *UAS*-*N*^*act*^ (**f**, **f**′) and *UAS*-*lgl*-*IR* (**g**, **g**′) remain similar as of wild-type brain (**e**, **e**′). **h** *UAS*-*N*^*act*^ and *UAS*-*lgl*-*IR* coexpressed brain shows massive expression of GFP in the optic lobes and in ventral nerve cord (arrowhead marks the expression of GFP in VNC). **h**′ The optic lobes and VNC of *N*^*act*^*/lgl*-*IR* brain shows extensive expression of MMP1 (arrow marks the expression of MMP1 in VNC). Images **e**″–**h**″ are merges of those in **e**–**e**′, **f**–**f**′, **g**–**g**′ and **h**–**h**′ with DAPI, respectively. All eye discs are oriented with dorsal to the left and anterior to the top position. Dorsal view of the brains is shown. Scale bars, 50 µm (**a**–**d**, **a**′–**d**′, **a**″–**d**″) and 100 µm (**e**–**h**, **e**′–**h**′, **e**″–**h**″)

Coexpression of both *lgl*-*IR* and *Notch*^*act*^ in the *Drosophila* eye discs using *ey*-*GAL4* dramatically induced overgrowth (Fig. 1d, d″) as compared to that of only *N*^*act*^ overexpressed (Fig. 1b, b″) or only *lgl*-*IR* overexpressed (Fig. 1c, c″) eye discs. To further describe the phenotype of *N*^*act*^*/lgl*-*IR* tumor, expression of Matrix metalloproteinase 1 (MMP1) was monitored. MMPs are enzymes with clear association to tumor cell invasion and cancer progression [14, 15]. Coexpression of *Notch*^*act*^ and *lgl*-*IR* resulted in massive upregulation of MMP1 expression throughout the entire eye disc (Fig. 1d′) as compared to that of only *N*^*act*^ or only *lgl*-*IR* (Fig. 1b′, c′). Further, we extended our observation into the brain since *ey*-*GAL* is mildly expressed in the brain also. Except endogenous expression, no MMP1 activation was observed in the *ey*-*GAL4* driven *lgl*-*IR* (Fig. 1g′) and *N*^*act*^ larval brain (Fig. 1f′). In case of *Notch*^*act*^*/lgl*-*IR* larval brain, excessive amount of GFP marked cells with enhanced MMP1 expression was observed in the optic lobes (Fig. 1h, h′). The increment in GFP and MMP1 expression was also found in the ventral nerve cord (VNC) of *Notch*^*act*^*/lgl*-*IR* larval brain (Fig. 1h, h′ marked with arrows). This indicates that the weak expression of *ey*-*GAL4* in VNC is also inducing MMP1 expression in *Notch*^*act*^*/lgl*-*IR* tissue. When we quantified the amount of GFP in upper region of VNC, a significant increment in the amount of GFP in *Notch*^*act*^*/lgl*-*IR* was found as compared to that of the controls (Additional file 2: Figure S1a). We also quantified the presence of MMP1 in the VNC of *Notch*^*act*^*/lgl*-*IR* (Additional file 2: Figure S1b), which clearly shows a significant increase as compared to that of the controls. Moreover, transcript levels of *mmp1* in the cephalic complex were also found to be upregulated in *Notch*^*act*^*/lgl*-*IR* tumor as compared to that of the controls (Additional file 2: Figure S1c).

In order to examine the cytoskeleton network and cell–cell adhesion, we marked the tissues with phalloidin and adherens junction marker proteins, Armadillo (Arm) and Cadherin (*D*E-Cad). The F-actin network marked by phalloidin revealed a defective actin cytoskeleton network in *N*^*act*^*/lgl*-*IR* tumor tissues compared to that of controls (Additional file 3: Figure S2). In the same way, the localization of *D*E-Cad and Arm were also deregulated in *N*^*act*^*/lgl*-*IR* tumorous eye discs (Additional file 4: Figure S3a–d, e–h). We, next, determined if neuronal differentiation was defective in *N*^*act*^*/lgl*-*IR* tumor using a neuronal marker, Elav that marks the differentiated neurons in eye disc and brain. Remarkably, coexpression of *N*^*act*^ and *lgl*-*IR* led to severe loss of Elav positive cells in the eye disc and abnormal expression of Elav in the optic lobes indicating an impaired neuronal differentiation (Additional file 4: Figure S3i–l, m–p).

In parallel, we also used dominant-negative version of Notch to see the effect of depletion of Notch signaling on *lgl*-*IR* tumors. Previously, expression of *mam*^*DN*^ in *lgl*^−^ tissues partially rescued the *lgl*^−^ mosaic adult eye phenotype [13]. Our analysis also found that reduction of Notch signaling partially rescued the phenotypes of *lgl* loss-of-function flies (Additional file 5: Figure S4). Thus, our analysis support the notion that the *lgl* loss-of-function wing phenotype is dependent on elevated Notch signaling, consistent with the previous study [13].

#### Involvement of JNK pathway in *N*^*act*^*/lgl*-*IR* tumor

**Fig. 2:**
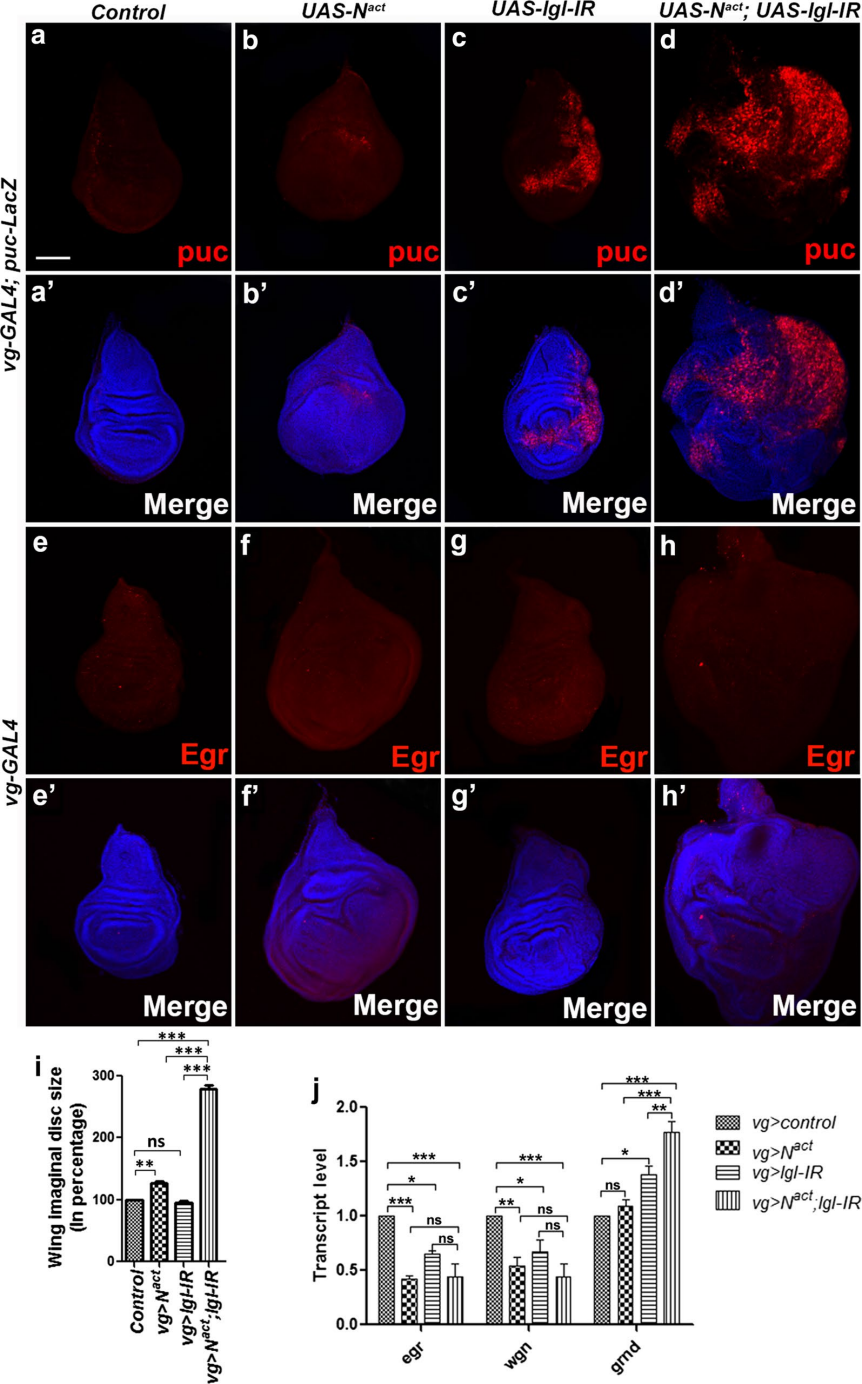
Activation of JNK signaling in *N*^*act*^*/lgl*-*IR* tumor. Fluorescent micrographs of wing imaginal discs are shown. **a** Endogenous expression of puc in *Drosophila* wing imaginal disc is shown. **b** Only *N*^*act*^ overexpression shows slight increment in *puc* expression. **c** Downregulation of *lgl* in wing disc induces *puc* expression. **d** A significant increase in *puc* expression was found, when *N*^*act*^ is coexpressed with *lgl*-*IR*. **a**′–**d**′ are the merge images of DAPI along with **a**–**d**, respectively. Images (**e**–**h**) show the expression of Egr in wild-type, *N*^*act*^, *lgl*-*IR*, and *N*^*act*^*/lgl*-*IR* wing discs, respectively. The expression of Egr remains unchanged in all genotypes. **e**′–**h**′ are the merge images of DAPI along with **e**–**h**. **i** Analyses of differences in wing disc size show that there is a significant increase in *N*^*act*^*/lgl*-*IR* wing imaginal disc size as compared to the wild-type, *N*^*act*^, and *lgl*-*IR* wing disc sizes. **j** Real-time PCR analysis was performed to estimate the transcript levels of JNK pathway ligand *egr* and its receptors *wgn* and *grnd* in *N*^*act*^*/lgl*-*IR* tumor. The transcript levels of *egr* and *wgn* in *N*^*act*^*/lgl*-*IR* were significantly reduced, when compared to that of wild-type; however no significant change in transcript levels were seen, when compared to that of only *N*^*act*^ and *lgl*-*IR*. Interestingly, *grnd* transcript levels were significantly upregulated in *N*^*act*^*/lgl*-*IR* tumor as compared to that of the wild-type, *N*^*act*^ and *lgl*-*IR* wing discs. qPCR was normalized with *rps17* and repeated for three times. Analysis of data was done using two-way ANOVA with Tukey’s multiple comparison test; data represents mean ± SEM (***p < 0.001; **p < 0.01; *p < 0.05 and ns *p *> 0.05). All wing discs are oriented with dorsal to the top and posterior to the right. Scale bars: 100 µm (**a**–**d**, **a**′–**d**′, **e**–**h**, **e**′–**h**′)

Previous studies in *Drosophila* have revealed that oncogenic Ras along with loss of *lgl* or *scrib* or *dlg* induces JNK signaling, which is crucial for tumor invasion [7, 16]. This prompted us to check the expression of Puckered (puc), a transcriptional target of JNK signaling and widely used to check the activation of JNK signaling. An enhancer trap allele, *puc*-*LacZ* [17] was used to monitor the activation of JNK signaling. Coexpression of both *N*^*act*^ and *lgl*-*IR* resulted in intense upregulation of *puc* throughout the wing disc (Fig. 2d), indicating the activation of JNK signaling in *N*^*act*^*/lgl*-*IR* tumor. We also observed a significant increase in size of the wing disc in *N*^*act*^ and *lgl*-*IR* coexpressed condition compared to that of the wild-type, only *N*^*act*^, and only *lgl*-*IR* wing discs (Fig. 2i).

To check the mode of activation of JNK signaling, we examined the transcript level expression of ligand *eiger* (*egr*), and its receptor *wengen (wgn),* in *N*^*act*^*/lgl*-*IR* tumor. *egr* and *wgn* transcript levels were found to be depleted in case of *N*^*act*^*/lgl*-*IR* tumor as compared to that of the controls (Fig. 2j). Recently, another member in tumor necrosis factor receptor superfamily, Grindelwald (Grnd), found to be associated with loss of cell polarity and neoplastic growth [18]. Interestingly, a significant upregulation of *grnd* transcripts in *N*^*act*^*/lgl*-*IR* tumor was found, when compared to that of the wild-type, only *N*^*act*^ and only *lgl*-*IR* tissues (Fig. 2j). We went on to check the protein level expression of Egr in *N*^*act*^*/lgl*-*IR* tumors. Immunostaining with anti-Egr antibody [19] revealed that there is no change in the level of Egr protein expression in *Nact/lgl*-*IR* tumor (Fig. 2h) as compared to that of the wild-type, only *N*^*act*^ and only *lgl*-*IR* tissues (Fig. 2e–g). As Egr is known to be also expressed by the tumor-associated hemocytes, leading to signaling activation [20], these immune cells may be in this case responsible for Grnd activation, but their poor adhesion to the tumor tissue may make them escape Immunofluorescence detection.

To further confirm the involvement of JNK signaling as a downstream event of *N*^*act*^*/lgl*-*IR* cooperation, we blocked JNK signaling in *N*^*act*^*/lgl*-*IR* tumor, and checked whether blocking JNK could affect the *N*^*act*^*/lgl*-*IR* tumor. The massive upregulation of MMP1 in *N*^*act*^*/lgl*-*IR* tumor (Additional file 6: Figure S5a) was drastically suppressed, when *bsk*-*DN* (a dominant negative allele of *Drosophila* JNK gene, *basket*) was expressed in the background (Additional file 6: Figure S5b). In addition, coexpression of *bsk*-*DN* with *N*^*act*^*; lgl*-*IR* resulted in a reduced wing disc size as compared to *N*^*act*^*/lgl*-*IR* overexpressed wing disc (Additional file 6: Figure S5c). These results indicate that JNK signaling may be involved in the tumorous overgrowth of *N*^*act*^*/lgl*-*IR* tissues.

#### *N*^*act*^*/lgl*-*IR* tumor induces cell death

**Fig. 3:**
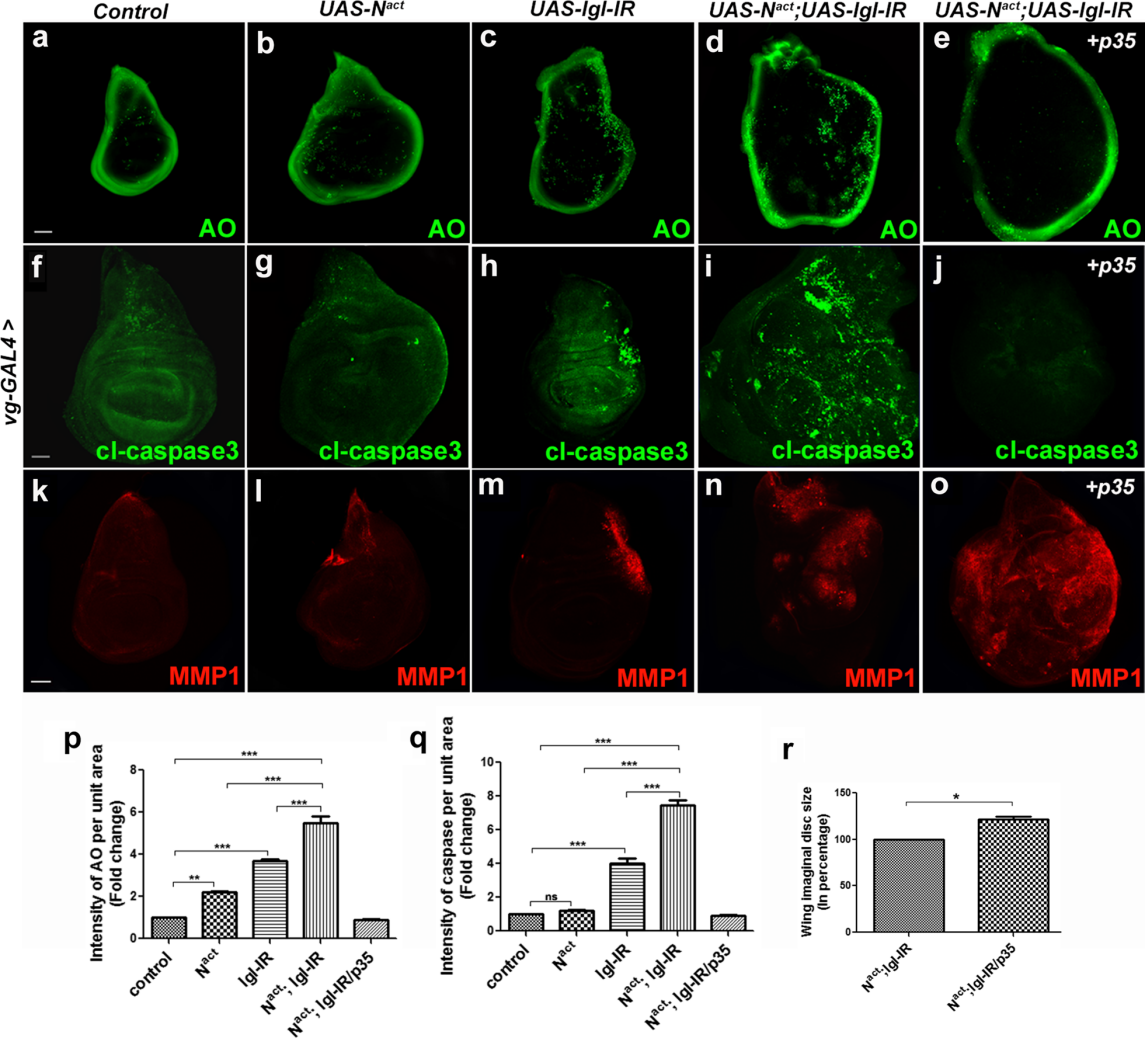
*N*^*act*^*/lgl*-*IR* tumor induces cell death. Fluorescent micrographs of wing imaginal discs are shown. Acridine orange-staining in wild-type (**a**), *N*^*act*^ over-expressed (**b**), *lgl*-*IR* over-expressed (**c**) and both *N*^*act*^*/lgl*-*IR* coexpressed (**d**) wing discs are shown. Expression of cleaved caspase 3 in wild-type (**f**), *N*^*act*^ over-expressed (**g**), *lgl*-*IR* over-expressed (**h**) are shown. **i** *N*^*act*^*/lgl*-*IR* coexpressed wing disc shows upregulation in cleaved caspase 3 expression. MMP1 expression in wild-type (**k**), *N*^*act*^ overexpressed (**l**), *lgl*-*IR* overexpressed (**m**) are shown. **n** *N*^*act*^*/lgl*-*IR* wing disc shows massive upregulation in MMP1 expression. Blocking cell death in *N*^*act*^*/lgl*-*IR* tissues by expressing *UAS*-*p35* leads to absence of acridine orange positive cells (**e**) and cleaved caspase-3 marked cells (**j**); whereas the expression of MMP1 is found to be unaltered (**o**). **p** Intensity per unit area for acridine orange shows that there is significant amount of upregulation of acridine orange-positive cells in *N*^*act*^*/lgl*-*IR* wing disc. **q** Intensity per unit area for caspase staining shows that the *N*^*act*^*/lgl*-*IR* wing disc contains significantly upregulated level of caspase activity than that of wild-type, *lgl*-*IR* and *N*^*act*^ wing discs. **r** Wing disc size analyses show that the disc size is increased when *p35* is expressed in the background of *N*^*act*^*/lgl*-*IR,* as compared to *N*^*act*^*/lgl*-*IR*. Analysis of data for intensity profiling was done using two-way ANOVA with Tukey’s multiple comparison test; data represents mean ± SEM (***p < 0.001; **p < 0.01 and ns *p *> 0.05. Analysis of data for wing disc size was done using Unpaired t test with Welch’s correction; data represents mean ± SEM *p < 0.05). All wing discs are oriented with dorsal to the top and posterior to the right. Scale bars: 100 µm for (**a**–**e**), and 50 µm for (**f**–**j**, **k**–**o**)

Eluding apoptosis is considered as one of the acquired capabilities of many types of cancer; however, studies also explain that elevated oncogenic signaling induces apoptosis or senescence [21]. When we checked the status of cell death in *N*^*act*^*/lgl*-*IR* tumor, we observed a significant amount of acridine orange (Compare Fig. 3d with a–c) and caspase positive cells (Compare Fig. 3i with f–h) indicating severe cell death. Since loss of *lgl* in a tissue known to induce cell competition to remove the unfit cells [22], dying cells in *N*^*act*^*/lgl*-*IR* tissue could be an indication of cell competition. To check the effect of cell death on overgrowth and MMP1 expression, we blocked cell death by expressing a caspase inhibitor, p35 (Fig. 3e, j). It was found that blocking cell death in *N*^*act*^*/lgl*-*IR* overexpressed condition did not obstruct MMP1 expression (Fig. 3o). Coexpression of *p35* with *N*^*act*^*/lgl*-*IR* resulted in an increased wing disc size as compared to *N*^*act*^*/lgl*-*IR* overexpressed wing disc (Fig. 3r). As the caspase inhibitor, p35 is known to block cell death [23], the increase in the tissue size is expected since blocking cell death in *N*^*act*^*/lgl*-*IR* tumor allowed more cells to overgrow that, in turn, increased the disc size.

### Discussion

In the present study, we unveil a cooperation of Notch with RNAi-mediated downregulation of a polarity cum tumor suppressor gene, *lgl* to promote tumor overgrowth. Our data, presented here, illustrate that coexpression of *N*^*act*^ and *lgl*-*IR* in *Drosophila* eye disc results in overgrowth, loss of positional clues and upregulation of MMP1 expression, which is less prevalent in only *N*^*act*^ overexpression or only *lgl*-*IR* overexpression. Earlier the loss of polarity gene *scribble* found to cooperate with Notch signaling to promote neoplastic overgrowth [2]. Another two independent studies of similar context show that oncogenic Ras cooperates with loss of cell polarity genes (*lgl, scrib, dlg*) to induce metastasis and secondary tumor formation at distant sites [7, 14]. Interestingly, we found that Notch synergizes with loss of *lgl* to promote tumorous overgrowth and elevated expression of MMP1, and inhibiting Notch signaling rescues the defects caused by loss of *lgl*. It indicates the potential function of Notch signaling during *lgl* mediated tumor development. Our data also show distorted epithelial integrity in *N*^*act*^*/lgl*-*IR* tumor that point towards epithelial to mesenchymal transition, where tightly joined epithelial cells with regularly spaced cell–cell junctions convert to mesenchymal cells which are of irregular shape without tight intracellular adhesion [24].

Further, we found upregulation of JNK signaling and its receptor Grindelwald in *N*^*act*^*/lgl*-*IR* tumor. Two previous studies have shown that Notch cooperates with two different proteins to induce proliferation and metastasis by the activation of JNK signaling in ligand-dependent and -independent manner [25, 26]. In case of *N*^*act*^*/lgl*-*IR* tumor, we show that the transcript levels of *egr* (ligand) and *wgn* (receptor) were not upregulated, whereas a significant upregulation of *grnd* transcripts in the *N*^*act*^*/lgl*-*IR* tumor was observed. Earlier the active form of Grnd has shown to activate JNK signaling in vivo [18]. Thus, in case of *N*^*act*^*/lgl*-*IR* tumor, JNK signaling might get activated through Grindelwald. Previously, it has been shown that JNK signaling can initiate tumor initiation and growth in Eiger-independent manner also [27].

Another most important hallmark of almost all types of cancer is the ability to evade apoptosis that, in turn, helps tumor cell population to increase in number [21]. In other similar tumor models such as *Ras*^*v12*^*/dlg*^−*/*−^, dying cells of *dlg*^−*/*−^ clones evade apoptosis in presence of oncogenic Ras, where JNK signaling switches its role from proapoptotic to progrowth [7]. In contrast, *Ras/scrib*^−*/*−^ and *Ras/lgl*^−*/*−^tumors were reported to show apoptosis [22, 28]. However, *Notch/scrib*^−*/*−^ tumor did not show the presence of apoptosis [29]. In our case, *N*^*act*^*/lgl*-*IR* tumor resulted in severe apoptosis along with strong overgrowth and MMP1 expression. These dying cells in *N*^*act*^*/lgl*-*IR* tumor might be the indication of cell competition as there is a strong proliferation and overgrowth. In case of *N*^*act*^*/scrib*^−*/*−^ tumor, Notch is giving growth advantage to *scrib*^−*/*−^ tissues by preventing cell death. However, in case of *N*^*act*^*/lgl*-*IR* wing discs, activation of Notch failed to restrict the cell death caused by loss of *lgl*; rather its activation induces further cell death. These differences indicate that although oncogenic cooperation with loss of cell polarity results in similar tumor cell migration but certain property like cell death occurs depending on the context.

## Limitations

- The present study is not the first one to show the cooperation between Notch and loss of cell polarity genes. Activated Notch is known to cooperate with another cell polarity gene, *scribble*, to induce neoplastic overgrowth.
- In the present study, experiments were performed using RNAi line of *lgl*, but not with the *lgl* loss-of-function mutants.

## Additional files

**Additional file 1.** Materials and methods.

**Additional file 2: Figure S1.** Quantification of GFP and MMP1 in the VNC of *N*^*act*^*/lgl*-*IR* tumor (**a**) GFP quantification in VNC shows a four-fold increment in the amount of GFP positive cells in *N*^*act*^*/lgl*-*IR* as compared to that of the wild-type, only *N*^*act*^ and *lgl*-*IR* overexpressed tissues. **b** MMP1 quantification in VNC shows around four-fold increase in *N*^*act*^*/lgl*-*IR*, whereas only *N*^*act*^ and *lgl*-*IR* overexpressed tissues show almost same level of MMP1 in VNC as of wild-type. **c** Real-Time PCR analysis shows significant increase in *mmp1* transcripts in the cephalic complex of *N*^*act*^*/lgl*-*IR* as compared to that of wild-type, only *N*^*act*^ or only *lgl*-*IR* tissues. Data was normalized to *rps17*. Analysis of data was done using One-way ANOVA with Tukey’s multiple comparison test; data represents mean ± SEM (***p < 0.001 and ns *p *> 0.05).

**Additional file 3: Figure S2.** *N*^*act*^*/lgl*-*IR* tumor leads to distorted actin cytoskeleton. Coexpression of *N*^*act*^ and *lgl*-*IR* causes distorted actin cytoskeleton organization (**d**) compared to that of wild-type (**a**), only *N*^*act*^ overexpressed (**b**) and only *lgl*-*IR* overexpressed condition (**c**). F-actin was marked using phalloidin. Scale bars: 10 µm (a-d).

**Additional file 4: Figure S3.** *N*^*act*^*/lgl*-*IR* shows hallmarks of migratory tumor. Fluorescent micrographs of eye imaginal discs and larval brains are shown. **a**, **a**′ Endogenous Cadherin and (**e**, **e**′) Armadillo localize at the adherens junctions and marks the photoreceptors in the *ey*-*GAL4/*+ eye imaginal discs. Morphogenetic furrow in **a** and **e** is marked with an arrow. Overexpression of *N*^*act*^ leads to overgrown discs and the localization pattern of Cadherin (**b**, **b**′) and Armadillo (**f**, **f**′) have been modified. Overexpression of *lgl*-*IR* results in distorted localization of Cadherin (**c**, **c**′) and Armadillo (**g**, **g**′). Coexpression of *N*^*act*^ and *lgl*-*IR* in eye imaginal disc causes complete deformation of Cadherin (**d**, **d**′) and Armadillo (**h**, **h**′) localization pattern. Images **a**′–**d**′, **e**′–**h**′ are higher magnification of the square region from **a**–**d**, **e**–**h**. **i** Expression of Elav, a marker for differentiated neurons in wild-type eye discs is shown. **j** Overexpression of *N*^*act*^ in eye disc shows increased expression of Elav, probably due to overproliferation of the disc. **k** *lgl*-*IR* over-expressed eye disc shows comparatively less Elav-positive cells. **l** Interestingly, *N*^*act*^ and *lgl*-*IR* coexpressed eye disc shows hardly any Elav-positive cells. Images **i**′, **j**′, **k**′ and **l**′ are merges of GFP along with **i**, **j**, **k** and **l**, respectively. Elav expression in the brains of *N*^*act*^ (**n**) and *lgl*-*IR* (**o**) driven by *ey*-*GAL4* is found to be similar to that of the wild-type brain (**m**). **p** Coexpression of *N*^*act*^ and *lgl*-*IR* resulted in an abnormal expression pattern of Elav, where clump like distribution is found in the optic lobes (marked with arrow). Images **m**′, **n**′, **o**′ and **p**′ are merges of GFP along with **m**, **n**, **o** and **p**, respectively. Scale bars: 50 µm (**a**–**d**, **e**–**h**, **i**–**l**, **i**′–**l**′), 5 µm (**a**′–**d**′, **e**′–**h**′) and 100 µm (**m**–**p**, **m**′–**p**′). All eye discs are oriented with dorsal to the left and anterior to the top. Ventral view of the brains is shown.

**Additional file 5: Figure S4.** Lowering the dose of Notch partially rescues *lgl*-*IR*-induced MMP1 expression and restores the adult wing. **a** MMP1 expression in wild-type wing disc is shown. **b** Overexpression of only *Notch*-*DN* did not induce expression of MMP1. **c** Overexpression of *lgl*-*IR* induces MMP1 expression in the wing disc. **d** Coexpression of *Notch*-*DN* in *lgl*-*IR* background partially rescues the expression of MMP1 caused by *lgl*-*IR* overexpression. **a**′, **b**′, **c**′ and **d**′ are merges of DAPI along with **a**, **b**, **c** and **d**, respectively. Moreover, Coexpression of *Notch*-*DN* with *lgl*-*IR* resulted in reduced wing disc size as compared to that of only overexpression of *lgl*-*IR* (**i**). **e** GFP marked vestigial domain in wing disc is shown. **e**′ is the merge image of DAPI along with (**e**). **f** Overexpression of *Notch*-*DN* resulted in held out wings with wing nicking phenotype. **g** Overexpression of *lgl*-*IR* using *vg*-*GAL4* led to necrotic lesions followed by deformation of adult wings. **h** Coexpression of *Notch*-*DN* with *lgl*-*IR* partially restored deformed adult wings. **j** Phenotype penetrance in adult flies is shown for each genotype; the phenotype observed in *Notch*-*DN* show 100% penetrance and around 70% *lgl*-*IR* flies showed deformed wings. In case of *Notch*-*DN; lgl*-*IR* flies, around 60% flies showed the depicted phenotype and, the rest of the flies showed less developed wings but they were not of the *lgl*-*IR* category. Analysis of data was done using One-way ANOVA with Tukey’s multiple comparison test; data represents mean ± SEM (***p < 0.001 and ns *p *> 0.05). All wing discs are oriented with dorsal to the top and posterior to the right. Scale bar: 50 µm (**a**–**d**, **a**′–**d**′).

**Additional file 6: Figure S5.** Inhibition of JNK pathway suppresses the *N*^*act*^*/lgl*-*IR* tumor growth and MMP1 expression. Fluorescent micrographs of wing imaginal discs are shown. **a** Overexpression of both *N*^*act*^ and *lgl*-*IR* in wing imaginal disc using *vg*-*GAL4* resulted in massive upregulation of MMP1. **b** Coexpression of *bsk*^*DN*^ in the background of *N*^*act*^ and *lgl*-*IR* resulted in the suppression of MMP1 expression. **a**″–**b**″ is the merge images of **a**–**a**′ and **b**–**b**″. **c** The *N*^*act*^*/lgl*-*IR* wing disc size was significantly reduced, when *bskDN* was expressed in the background. Analysis of data was done using Unpaired t test with Welch’s correction; data represents mean ± SEM **p < 0.01). All wing discs are oriented with dorsal to the top and posterior to the left. Scale bar: 50 µm (a–a″, b–b″).

## Abbreviations

scrib: scribble
lgl: lethal giant larvae
dlg: discs large
N^act^: activated Notch
lgl-IR: lgl-RNAi
MMP1: matrix metalloproteinase
VNC: ventran nerve cord
ey-GAL4: eyeless GAL4
Arm: armadillo
*D*E-Cad: *Drosophila* E-Cadherin
Notch-DN: Notch dominant negative
puc: puckered
egr: eiger
wgn: wengen
grnd: grindelwald
bsk-DN: basket-dominant negative
