# Supplementary material for: Notch signals modulate lgl mediated tumorigenesis by the activation of JNK signaling
Source: BMC Res Notes. 2018 Apr 16;11:247. doi: 10.1186/s13104-018-3350-5 (PMC5902968; doi:10.1186/s13104-018-3350-5)
Supplement: Supplementary file 1 — Additional file 1. Materials and methods. [file 13104_2018_3350_MOESM1_ESM.docx]

**Additional File legends**

**Additional File 1 - Materials and Methods**

***Drosophila* stocks and genetics**

The following *Drosophila* stocks were used: *UAS-lgl-IR* (BL# 35773), *ey-GAL4:UAS-GFP, vg-GAL4, UAS-p35, UAS-bsk^DN^* stocks were obtained from Bloomington stock center. *UAS-Notch^act^* and *UAS-Notch-DN* flies were kindly provided by Spyros Artavanis-Tsakonas. *puc-lacZ* stock was provided by Estee Kurant. *ptc-GAL4,UAS-GFP;GAL80^ts^* stock was obtained from the laboratory of Florenci Serras. The combination stocks *UAS-Notch^act^*; *UAS-lgl-IR, UAS-Notch-DN*;*UAS-lgl-IR, vg-GAL4;puc-LacZ, vg-GAL4;UAS-p35, UAS-GFP;UAS-lgl-IR, and vg-GAL4; UAS-bsk^DN^* were generated by appropriate genetic crosses. To induce the expression of the genes in the ptc domain of the wing disc, we used the temperature-sensitive GAL80 construct with UAS/GAL4 system. At lower temperature (18°C), GAL80 inhibits the function of GAL4, and at a higher temperature (29°C), GAL80^ts^ is inactive. Thus, it is unable to inhibit GAL4 at higher temperature, which in turn activates the expression of the genes under the UAS promoter. In our experiment, crosses were set and kept at 18°C for 6 days, and the larvae were then shifted to 29°C and kept until the third instar larvae develops.

**Immunocytochemistry and confocal microscopy**

Immunostaining was performed as previously described [1]. Primary antibodies used were mouse anti-MMP1 (1:100), mouse anti-Armadillo (1:100), rat anti-*D*E-Cadherin (1:100), rat anti-Elav (1:200), mouse anti-Dlg (1:100) (all from Developmental Studies Hybridoma Bank), rabbit anti-cleaved caspase 3 (1:50, Cell Signaling Technologies), mouse anti-β gal (1:100, Promega), TRIT-C conjugated phalloidin (1:100, Sigma) and mouse anti-Egr (1:100, kind gift from Konrad Basler). Secondary antibodies used were goat anti-mouse Alexa-555 (1:200), goat anti-rat Alexa-555 (1:200), anti-rabbit Alexa-555 (1:200) (Molecular Probes, Eugene, OR). The images were taken using Carl Zeiss 780 laser scanning confocal microscopy. All images were processed and assembled using Adobe photoshop 7.0.

**Acridine orange staining**

Wing imaginal discs from larvae of desired genotypes were dissected in ice-cold 1X PBS and incubated in Acridine orange (1µg/ml) solution (Sigma) in PBS for 2 minutes at room temperature. The tissues were quickly washed for 2 times and mounted in PBS followed by observation and imaging in Nikon Eclipse Ni fluorescence microscope.

**Whole fly imaging, measurement of wing imaginal disc size, and GFP quantification**

Anesthetized adult flies were placed on a glass slide, and images were captured using Leica MZ10F microscope. For measuring the disc size, wing imaginal discs from L3 larvae were dissected and photographed at a constant magnification. The whole area of each disc was measured in arbitrary units using ImageJ software and normalized to wild-type as a percentage. These normalized data were then plotted using GraphPad Prism5. The imaginal disc size was measured in a two-dimensional way. The fluorescence signals for GFP and MMP1 in the required area were quantified using ImageJ software; intensity of fluorescence was measured in parallel with wild-type tissues and normalized with the controls.

**RNA isolation and Real-time PCR**

Total RNA from 50 cephalic complex and 100 wing imaginal discs of *Drosophila* third instar larvae was extracted using TRI reagent as per manufacturer’s (Sigma) instructions. RNA was resuspended in nuclease-free water and quantified using a nanodrop. Equal amount of RNA for each data set were incubated with RNase free DNase (Roche) for 30 min at 37˚C to remove genomic DNA contamination. First-strand cDNA was synthesized from 2µg of total RNA using High Capacity cDNA Reverse Transcription kit as per manufacturer’s instructions (Applied biosystems). Quantitative real-time PCR was performed as previously described [2]. The sequences of primers are available on request.

**References for Additional File1**

1. Mukherjee A, Veraska A, Bauer A, Rosse C, Camonis J et al., Regulation of notch signaling by non-visual beta arrestin. Nat. Cell Biol. 2005;7:1191–1201.
2. Sachan N, Mishra AK, Mutsuddi M, Mukherjee A. Chip physically interacts with Notch and their stoichiometry is critical for Notch function in wing development and cell proliferation in *Drosophila.* [Biochim Biophys Acta*.*](http://www.ncbi.nlm.nih.gov/pubmed/25597954) 2015;1850:802-12.

**Additional File 2: Figure S1**

**Quantification of GFP and MMP1 in the VNC of *N^act^/lgl-IR* tumor** (**a**) GFP quantification in VNC shows a four-fold increment in the amount of GFP positive cells in *N^act^/lgl-IR* as compared to that of the wild-type, only *N^act^* and *lgl-IR* overexpressed tissues. (**b**) MMP1 quantification in VNC shows around four-fold increase in *N^act^/lgl-IR***,** whereas only *N^act^* and *lgl-IR* overexpressed tissues show almost same level of MMP1 in VNC as of wild-type. (**c**) Real-Time PCR analysis shows significant increase in *mmp1* transcripts in the cephalic complex of *N^act^/lgl-IR* as compared to that of wild-type, only *N^act^* or only *lgl-IR* tissues. Data was normalized to *rps17*. Analysis of data was done using One-way ANOVA with Tukey’s multiple comparison test; data represents mean ± SEM (****p*<0.001 and ns *p*>0.05).

**Additional file 3: Figure S2**

***N^act^/lgl-IR* tumor leads to distorted actin cytoskeleton.** Coexpression of *N^act^* and *lgl-IR* causes distorted actin cytoskeleton organization (**d**) compared to that of wild-type (**a**), only *N^act^* overexpressed (**b**) and only *lgl-IR* overexpressed condition(**c**). F-actin was marked using phalloidin. Scale bars: 10µm (a-d).

**Additional file 4: Figure S3**

***N^act^/lgl-IR* shows hallmarks of migratory tumor.** Fluorescent micrographs of eye imaginal discs and larval brains are shown. (**a, a’**) Endogenous Cadherin and (**e, e’**) Armadillo localize at the adherens junctions and marks the photoreceptors in the *ey-GAL4/+* eye imaginal discs. Morphogenetic furrow in a and e is marked with an arrow. Overexpression of *N^act^* leads to overgrown discs and the localization pattern of Cadherin (**b**, **b’**) and Armadillo (**f**, **f’**) have been modified. Overexpression of *lgl-IR* results in distorted localization of Cadherin (**c**, **c’**) and Armadillo (**g, g’**). Coexpression of *N^act^* and *lgl-IR* in eye imaginal disc causes complete deformation of Cadherin (**d**, **d’**) and Armadillo (**h**, **h’**) localization pattern. Images a’-d’, e’-h’ are higher magnification of the square region from a-d, e-h. (**i**) Expression of Elav, a marker for differentiated neurons in wild-type eye discs is shown. (**j**) Overexpression of *N^act^* in eye disc shows increased expression of Elav, probably due to overproliferation of the disc. (**k**) *lgl-IR* over-expressed eye disc shows comparatively less Elav-positive cells. (**l**) Interestingly, *N^act^* and *lgl-IR* coexpressed eye disc shows hardly any Elav-positive cells. Images **i’, j’, k’** and **l’** are merges of GFP along with **i, j, k** and **l,** respectively. Elav expression in the brains of *N^act^* (**n**) and *lgl-IR* (**o**) driven by *ey-GAL4* is found to be similar to that of the wild-type brain (**m**). (**p**) Coexpression of *N^act^* and *lgl-IR* resulted in an abnormal expression pattern of Elav, where clump like distribution is found in the optic lobes (marked with arrow). Images **m’, n’, o’** and **p’** are merges of GFP along with **m, n, o** and **p,** respectively. Scale bars: 50µm (a-d, e-h, i-l, i’-l’), 5µm (a’-d’, e’- h’) and 100µm (m-p, m’-p’). All eye discs are oriented with dorsal to the left and anterior to the top. Ventral view of the brains is shown.

**Additional file 5: Figure S4**

**Lowering the dose of Notch partially rescues *lgl-IR*-induced MMP1 expression and restores the adult wing.** (**a**) MMP1 expression in wild-type wing disc is shown. (**b**) Overexpression of only *Notch-DN* did not induce expression of MMP1. (**c**) Overexpression of *lgl-IR* induces MMP1 expression in the wing disc. (**d**) Coexpression of *Notch-DN* in *lgl-IR* background partially rescues the expression of MMP1 caused by *lgl-IR* overexpression. **a’, b’, c’** and **d’** are merges of DAPI along with **a, b, c** and **d,** respectively. Moreover, Coexpression of *Notch-DN* with *lgl-IR* resulted in reduced wing disc size as compared to that of only overexpression of *lgl-IR* (**i**). (**e**) GFP marked vestigial domain in wing disc is shown. **e’** is the merge image of DAPI along with (**e**). (**f**) Overexpression of *Notch-DN* resulted in held out wings with wing nicking phenotype. (**g**) Overexpression of *lgl-IR* using *vg-GAL4* led to necrotic lesions followed by deformation of adult wings. (**h**) Coexpression of *Notch-DN* with *lgl-IR* partially restored deformed adult wings. (**j**) Phenotype penetrance in adult flies is shown for each genotype; the phenotype observed in *Notch-DN* show 100% penetrance and around 70% *lgl-IR* flies showed deformed wings. In case of *Notch-DN; lgl-IR* flies, around 60% flies showed the depicted phenotype and, the rest of the flies showed less developed wings but they were not of the *lgl-IR* category. Analysis of data was done using One-way ANOVA with Tukey’s multiple comparison test; data represents mean ± SEM (****p*<0.001 and ns *p*>0.05). All wing discs are oriented with dorsal to the top and posterior to the right. Scale bar: 50µm (a-d, a’-d').

**Additional file 6: Figure S5**

**Inhibition of JNK pathway suppresses the *N^act^/lgl-IR* tumor growth and MMP1 expression.**

Fluorescent micrographs of wing imaginal discs are shown. (**a**) Overexpression of both *N^act^* and *lgl-IR* in wing imaginal disc using *vg-GAL4* resulted in massive upregulation of MMP1. (**b**) Coexpression of *bsk^DN^* in the background of *N^act^* and *lgl-IR* resulted in the suppression of MMP1 expression. **a”-b”** is the merge images of **a**-**a’** and **b-b”**. (**c**) The *N^act^/ lgl-IR* wing disc size was significantly reduced, when *bskDN* was expressed in the background. Analysis of data was done using Unpaired t test with Welch’s correction; data represents mean ± SEM ***p*<0.01). All wing discs are oriented with dorsal to the top and posterior to the left. Scale bar: 50µm (a-a”, b-b”).
