# Supplementary figures and images for: Notch signals modulate lgl mediated tumorigenesis by the activation of JNK signaling
Source: BMC Res Notes. 2018 Apr 16;11:247. doi: 10.1186/s13104-018-3350-5 (PMC5902968; doi:10.1186/s13104-018-3350-5)

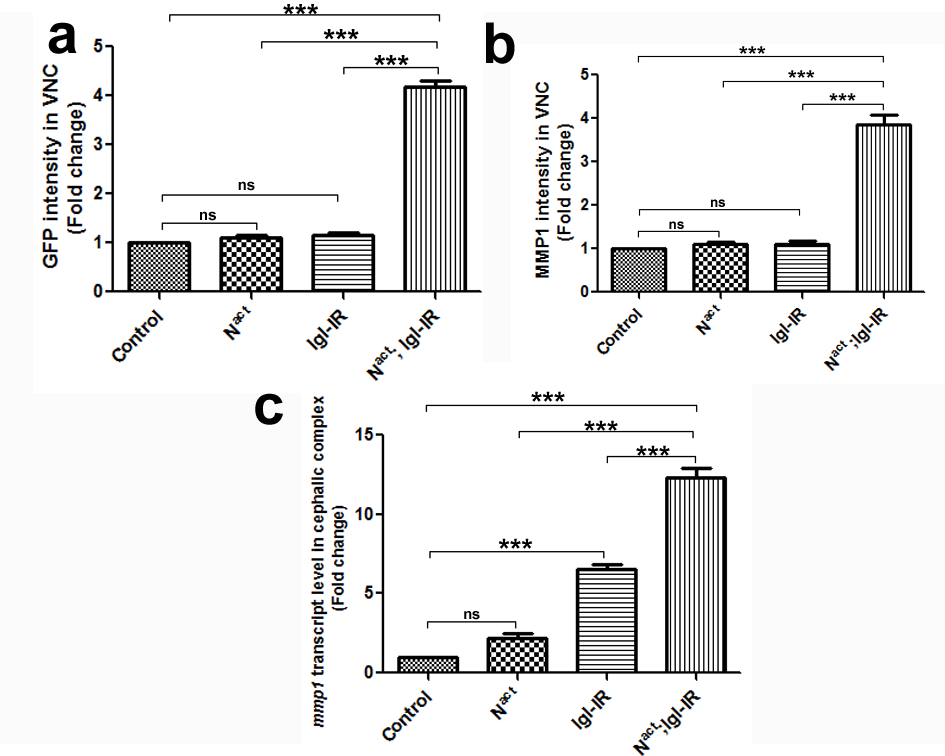

Supplement: Supplementary file 2 — Additional file 2: Figure S1. Quantification of GFP and MMP1 in the VNC of Nact/lgl-IR tumor (a) GFP quantification in VNC shows a four-fold increment in the amount of GFP positive cells in Nact/lgl-IR as compared to that of the wild-type, only Nact and lgl-IR overexpressed tissues. b MMP1 quantification in VNC shows around four-fold increase in Nact/lgl-IR, whereas only Nact and lgl-IR overexpressed tissues show almost same level of MMP1 in VNC as of wild-type. c Real-Time PCR analysis shows significant increase in mmp1 transcripts in the cephalic complex of Nact/lgl-IR as compared to that of wild-type, only Nact or only lgl-IR tissues. Data was normalized to rps17. Analysis of data was done using One-way ANOVA with Tukey’s multiple comparison test; data represents mean ± SEM (***p < 0.001 and ns p > 0.05). [file 13104_2018_3350_MOESM2_ESM.tif]

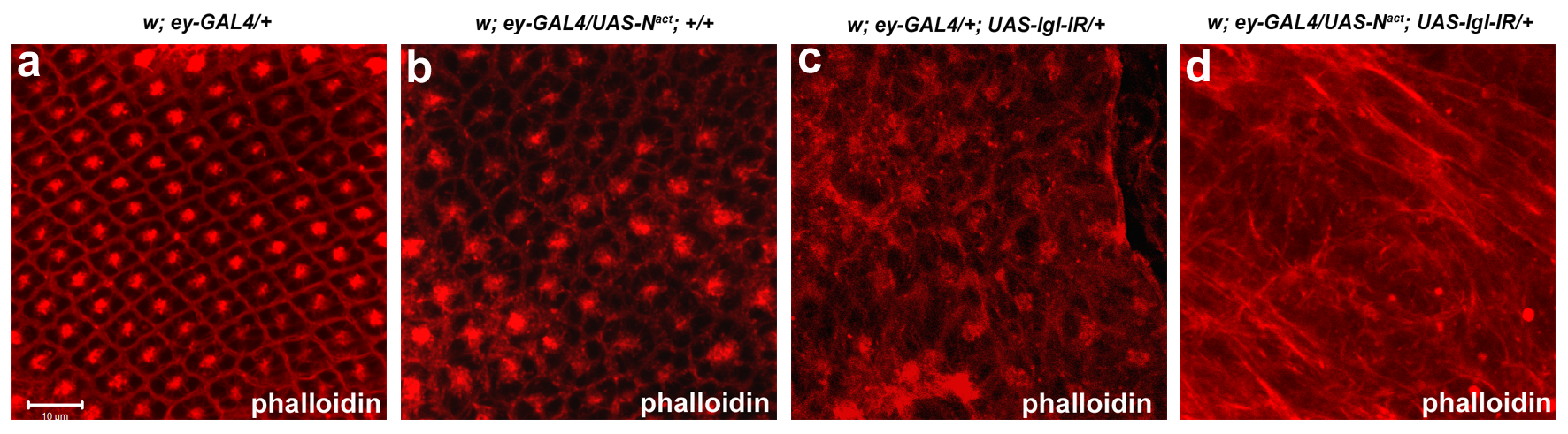

Supplement: Supplementary file 3 — Additional file 3: Figure S2. Nact/lgl-IR tumor leads to distorted actin cytoskeleton. Coexpression of Nact and lgl-IR causes distorted actin cytoskeleton organization (d) compared to that of wild-type (a), only Nact overexpressed (b) and only lgl-IR overexpressed condition (c). F-actin was marked using phalloidin. Scale bars: 10 µm (a-d). [file 13104_2018_3350_MOESM3_ESM.tif]

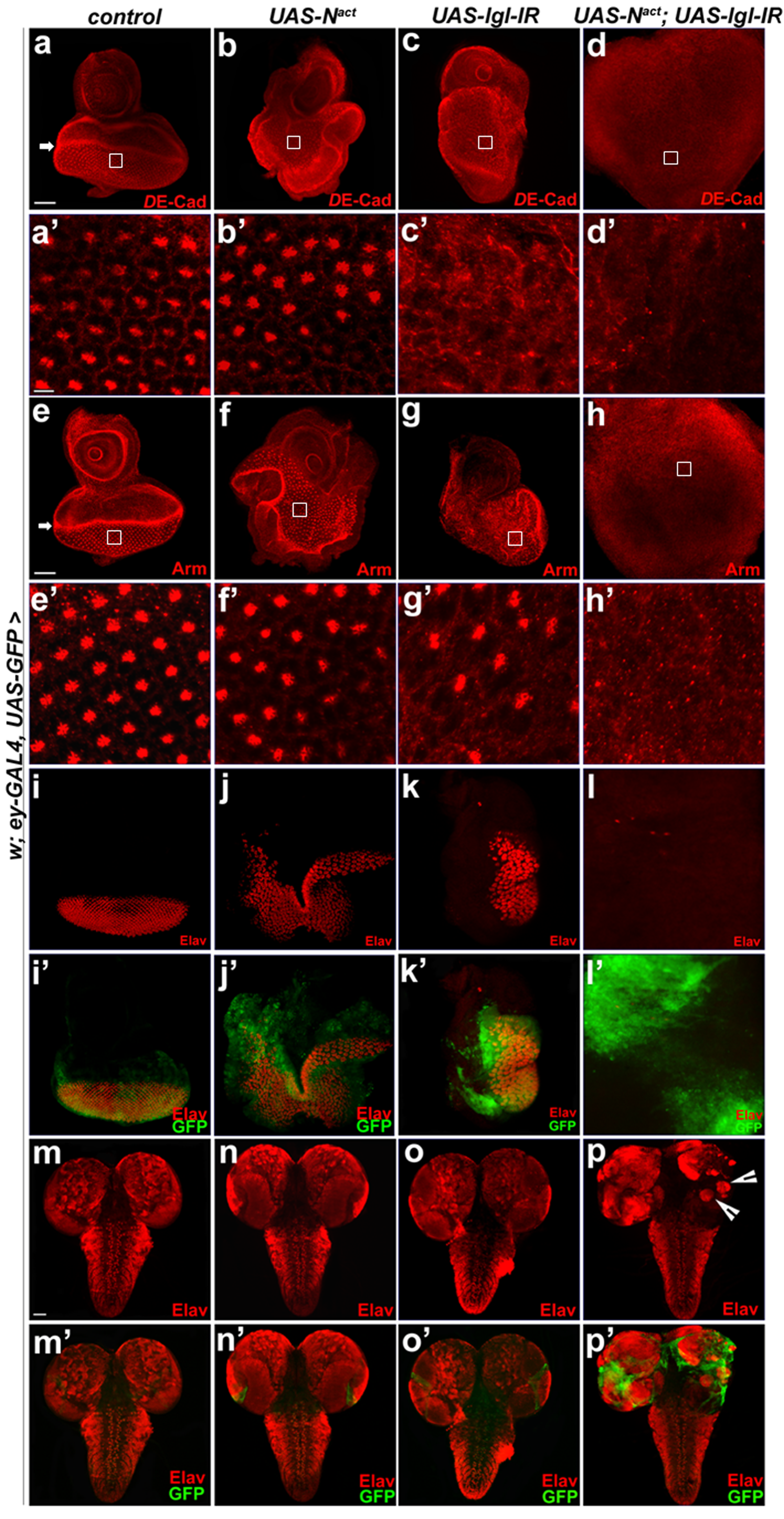

Supplement: Supplementary file 4 — Additional file 4: Figure S3. Nact/lgl-IR shows hallmarks of migratory tumor. Fluorescent micrographs of eye imaginal discs and larval brains are shown. a, a′ Endogenous Cadherin and (e, e′) Armadillo localize at the adherens junctions and marks the photoreceptors in the ey-GAL4/+ eye imaginal discs. Morphogenetic furrow in a and e is marked with an arrow. Overexpression of Nact leads to overgrown discs and the localization pattern of Cadherin (b, b′) and Armadillo (f, f′) have been modified. Overexpression of lgl-IR results in distorted localization of Cadherin (c, c′) and Armadillo (g, g′). Coexpression of Nact and lgl-IR in eye imaginal disc causes complete deformation of Cadherin (d, d′) and Armadillo (h, h′) localization pattern. Images a′–d′, e′–h′ are higher magnification of the square region from a–d, e–h. i Expression of Elav, a marker for differentiated neurons in wild-type eye discs is shown. j Overexpression of Nact in eye disc shows increased expression of Elav, probably due to overproliferation of the disc. k lgl-IR over-expressed eye disc shows comparatively less Elav-positive cells. l Interestingly, Nact and lgl-IR coexpressed eye disc shows hardly any Elav-positive cells. Images i′, j′, k′ and l′ are merges of GFP along with i, j, k and l, respectively. Elav expression in the brains of Nact (n) and lgl-IR (o) driven by ey-GAL4 is found to be similar to that of the wild-type brain (m). p Coexpression of Nact and lgl-IR resulted in an abnormal expression pattern of Elav, where clump like distribution is found in the optic lobes (marked with arrow). Images m′, n′, o′ and p′ are merges of GFP along with m, n, o and p, respectively. Scale bars: 50 µm (a–d, e–h, i–l, i′–l′), 5 µm (a′–d′, e′–h′) and 100 µm (m–p, m′–p′). All eye discs are oriented with dorsal to the left and anterior to the top. Ventral view of the brains is shown. [file 13104_2018_3350_MOESM4_ESM.tif]

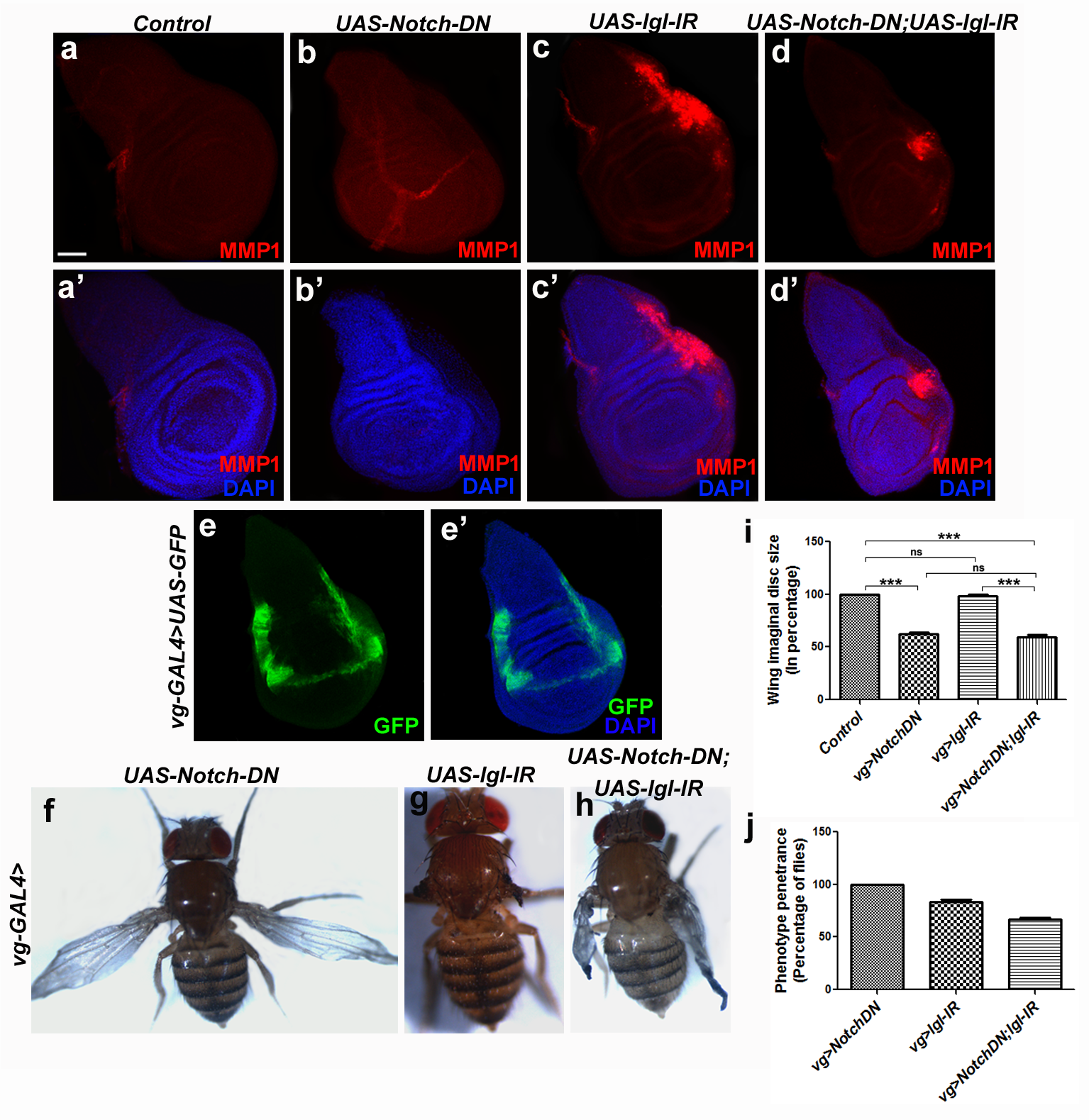

Supplement: Supplementary file 5 — Additional file 5: Figure S4. Lowering the dose of Notch partially rescues lgl-IR-induced MMP1 expression and restores the adult wing. a MMP1 expression in wild-type wing disc is shown. b Overexpression of only Notch-DN did not induce expression of MMP1. c Overexpression of lgl-IR induces MMP1 expression in the wing disc. d Coexpression of Notch-DN in lgl-IR background partially rescues the expression of MMP1 caused by lgl-IR overexpression. a′, b′, c′ and d′ are merges of DAPI along with a, b, c and d, respectively. Moreover, Coexpression of Notch-DN with lgl-IR resulted in reduced wing disc size as compared to that of only overexpression of lgl-IR (i). e GFP marked vestigial domain in wing disc is shown. e′ is the merge image of DAPI along with (e). f Overexpression of Notch-DN resulted in held out wings with wing nicking phenotype. g Overexpression of lgl-IR using vg-GAL4 led to necrotic lesions followed by deformation of adult wings. h Coexpression of Notch-DN with lgl-IR partially restored deformed adult wings. j Phenotype penetrance in adult flies is shown for each genotype; the phenotype observed in Notch-DN show 100% penetrance and around 70% lgl-IR flies showed deformed wings. In case of Notch-DN; lgl-IR flies, around 60% flies showed the depicted phenotype and, the rest of the flies showed less developed wings but they were not of the lgl-IR category. Analysis of data was done using One-way ANOVA with Tukey’s multiple comparison test; data represents mean ± SEM (***p < 0.001 and ns p > 0.05). All wing discs are oriented with dorsal to the top and posterior to the right. Scale bar: 50 µm (a–d, a′–d′). [file 13104_2018_3350_MOESM5_ESM.tif]

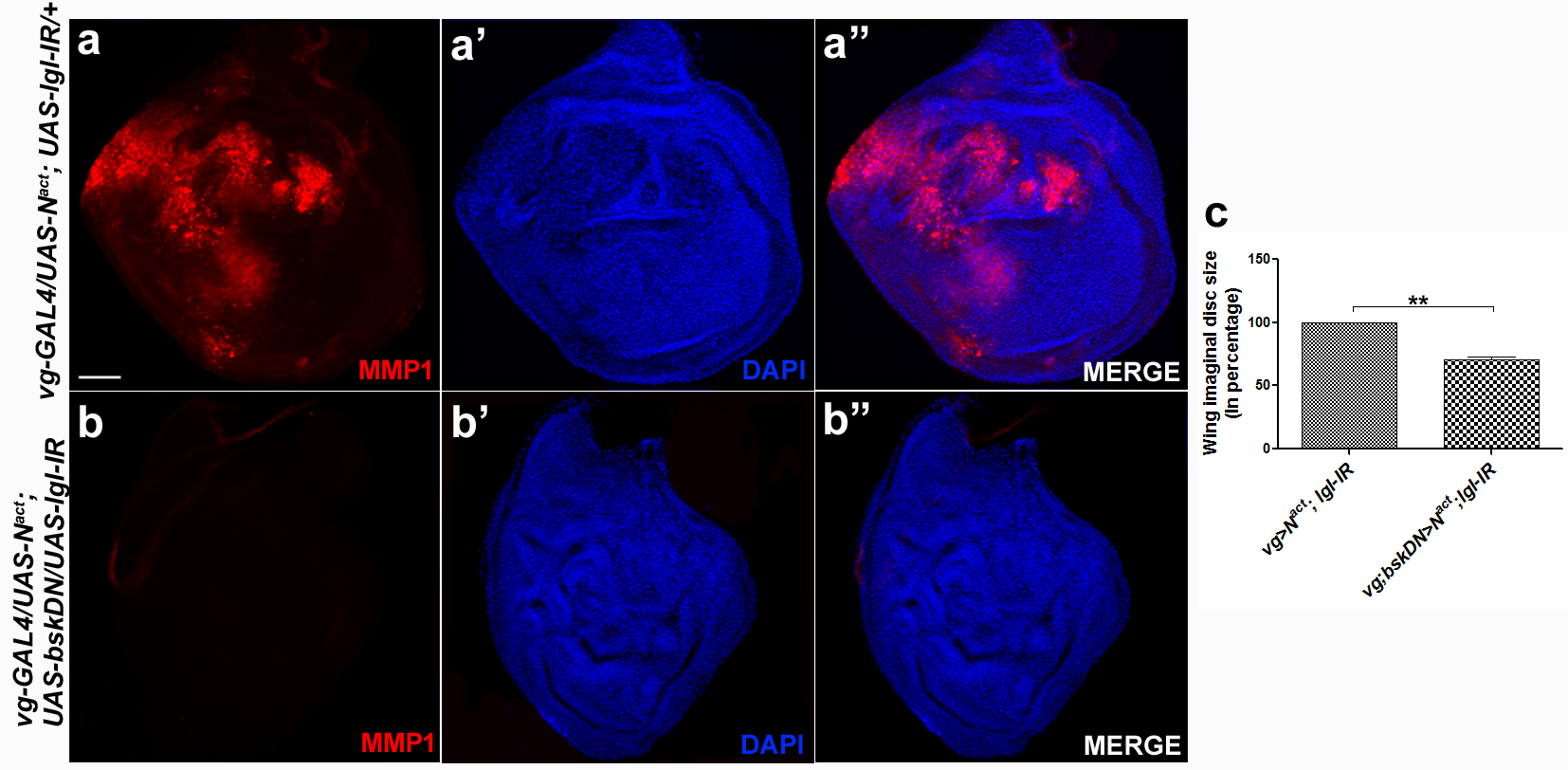

Supplement: Supplementary file 6 — Additional file 6: Figure S5. Inhibition of JNK pathway suppresses the Nact/lgl-IR tumor growth and MMP1 expression. Fluorescent micrographs of wing imaginal discs are shown. a Overexpression of both Nact and lgl-IR in wing imaginal disc using vg-GAL4 resulted in massive upregulation of MMP1. b Coexpression of bskDN in the background of Nact and lgl-IR resulted in the suppression of MMP1 expression. a″–b″ is the merge images of a–a′ and b–b″. c The Nact/lgl-IR wing disc size was significantly reduced, when bskDN was expressed in the background. Analysis of data was done using Unpaired t test with Welch’s correction; data represents mean ± SEM **p < 0.01). All wing discs are oriented with dorsal to the top and posterior to the left. Scale bar: 50 µm (a–a″, b–b″). [file 13104_2018_3350_MOESM6_ESM.tif]
